# Challenges in Diagnosing and Managing Severe Hypernatremia in Patients With Major Depressive Disorder: A Case Report

**DOI:** 10.7759/cureus.64281

**Published:** 2024-07-10

**Authors:** Hong Thoai Nguyen, Ayah Alkhidir, Yanira V Lagos, Pham Thao Vy Le, Lac Han Nguyen

**Affiliations:** 1 Internal Medicine, Ascension Saint Joseph Hospital, Chicago, USA; 2 Cardiology, Methodist Hospital, Merrillville, USA; 3 Internal Medicine, University of Texas at Tyler, Tyler, USA

**Keywords:** correction rate, dextrose 5%, thirst mechanism, hypernatremia, major depressive disorder (mdd)

## Abstract

Hypernatremia, characterized by a plasma sodium concentration above 145 mmol/L, is frequently observed in critically ill patients, often due to factors such as gastrointestinal losses, dehydration, and diabetes insipidus. Psychiatric patients, particularly those with major depressive disorder, are also at risk of developing hypernatremia due to abnormalities in thirst sensation, mineralocorticoid excess, or medication side effects. Severe hypernatremia in psychiatric patients is associated with a high mortality rate, presenting challenges in diagnosis and management. The treatment of chronic hypernatremia (>48 hours) typically involves administering isotonic saline to hypovolemic patients until normalization of vital signs, followed by dextrose 5% in water (D5W) based on water deficit and losses. The goal is to decrease plasma sodium by 8-10 mmol/day. Acute hypernatremia (<48 hours) is corrected with a plasma sodium reduction of 1 mmol/L/hour in the first six to eight hours. While there are no clear guidelines for sodium correction in severe hypernatremia, the literature suggests a safe correction rate of 8-10 mmol/day for chronic hypernatremia and 1 mmol/L/hour for acute cases. In a specific case, a 51-year-old female with severe depression and reduced oral intake was admitted. She exhibited signs of dehydration and was found to have severe hypernatremia (191 mmol/L) with acute kidney injury. Treatment involved D5W, followed by D5W/half-normal saline at 150 mL/hr. Within 24 hours, her plasma sodium decreased to 178 mmol/L and gradually normalized to 143 mmol/L without neurological complications. This case highlights the challenges and underscores the importance of early recognition and management of severe hypernatremia in psychiatric patients. The primary treatment approach addresses water deficits and losses and administers D5W. Recent findings suggest that rapid correction of the condition is acceptable.

## Introduction

Hypernatremia is a commonly encountered electrolyte disorder in clinical practice, particularly among critically ill patients. It is characterized by a plasma sodium concentration exceeding 145 mmol/L [1,2] and can stem from various factors such as gastrointestinal and cutaneous losses, mechanical ventilation, diuresis, diabetes insipidus, and dehydration. Vulnerable populations, including infants, psychiatric patients, and the elderly, are at heightened risk of developing hypernatremia [3-5]. Psychiatric patients may exhibit both hyponatremia due to psychogenic polydipsia and hypernatremia due to psychogenic adipsia. The former involves excessive voluntary water intake, while the latter signifies inappropriately reduced or absent thirst sensations. Severe hypernatremia is associated with a high mortality rate, reaching 50-70% in adults [6,7] and leading to causes such as multiorgan failure, circulatory failure, and septic shock [8]. Although hypernatremia is less common among psychiatric patients, its diagnosis and management within this patient subgroup can prove challenging. This case study highlights the difficulties encountered in diagnosing and managing severe hypernatremia in a patient with major depressive disorder at a community hospital.

## Case presentation

A 51-year-old African-American woman with a medical history notable for major depressive disorder, schizophrenia, anemia, and sickle cell trait was initially admitted to our hospital’s behavioral health unit due to severe depression and reduced oral intake. Her symptoms of schizophrenia were effectively managed prior to hospitalization. However, she has been experiencing numerous stressors in her life over the past few weeks. Despite a previous admission to the psychiatric unit for major depressive disorder and multiple visits to the emergency room with somatic symptoms, all examinations yielded no significant findings. A laboratory analysis conducted seven days before admission showed a serum sodium level of 142 mmol/L, although the patient declined further testing upon admission.

Upon admission, the patient expressed feelings of depression, including a depressed mood and anhedonia. She was not interested in doing her hobbies. She felt guilt and worthlessness. Her appetite was reduced, leading to poor oral intake and a lack of sleep, but she denied having thoughts of suicidal or homicidal ideation. The patient also denied recent medical conditions, alcohol, or drug use but mentioned having dark-colored urine. Subsequent urine analysis confirmed a urinary tract infection, for which the patient was treated with ceftriaxone for five days. During her time in the behavioral health unit, the patient was administered scheduled quetiapine 25 mg once a day and as-needed medications, including benztropine 1 mg twice a day, diphenhydramine 50 mg twice a day, olanzapine 5 mg three times a day, lorazepam 1 mg once a day, and trazodone 50 mg nightly. Over four days, the patient became increasingly withdrawn, refusing to take oral medication, eat, or undergo laboratory tests.

On the fifth day of hospitalization, she displayed signs of dehydration, including tachycardia, dry mucous membranes, and decreased urine output, prompting a transfer to the medical floor for dehydration management. Laboratory analysis revealed severe hypernatremia with a plasma sodium concentration of 191 mmol/L, as well as other electrolyte abnormalities and acute kidney injury (Table 1). Upon transfer to the intensive care unit, the patient remained lethargic but responsive, requiring treatment with a 1-liter bolus of dextrose 5% in water (D5W) followed by D5W/1/2 NS at 150 ml/hr. Within 24 hours, her plasma sodium concentration was corrected by 13 mmol/L (Table 1). Despite subsequent adjustments in intravenous fluid therapy and improvements in plasma sodium concentration, the patient remained lethargic and refused oral intake.

**Table 1:** Blood work before admission and during admission The patient’s labs were not available on the day of admission due to her refusion.

Component	Reference range	Seven days before admission	Five days after admission	Six days after admission (24 hours after treatment)	Ten days after admission	Twelve days after admission
Blood urea nitrogen (mg/dl)	7-25	12	66	62	13	9
Creatinine (mg/dl)	0.6-1.2	0.56	1.95	2.04	0.73	0.64
Sodium (mmol/L)	133-144	142	191	178	156	143
Potassium (mmol/L)	3.5-5.2	3.7	3.2	3.1	3.5	3.2
Chloride (mmol/L)	98-107	106	157	147	123	111

Nevertheless, on the fifth day of treatment in the intensive care unit, the patient’s cognitive function showed signs of improvement, and her acute kidney injury resolved. Her plasma sodium concentration decreased to 156 mmol/L, and she demonstrated good tolerance for oral intake. Subsequently, on the seventh day, with her plasma sodium concentration at 143 mmol/L, intravenous fluids were ceased, and she was relocated to the medical floor for ongoing care and support to ensure continued oral intake.

## Discussion

Hypernatremia is typically diagnosed when the sodium concentration in the plasma exceeds 145 mmol/L [9]. The causes of hypernatremia are categorized based on the patient’s volume status. Volume overload may occur due to excessive salt intake, administration of hypertonic saline, errors during hemodialysis, and primary aldosteronism. In euvolemic and hypovolemic states, hypernatremia can result from gastrointestinal losses (such as diarrhea and vomiting), cutaneous loss (through sweating and burns), mechanical ventilation, osmotic diuresis (as seen with mannitol use) [10], administration of loop diuretics, hypodipsia, adipsia, central diabetes insipidus (caused by conditions like brain injury, surgery, tumors, or infections), and nephrogenic diabetes insipidus (associated with conditions like hypercalcemia, hypokalemia, and use of lithium).

In cases of acute hypernatremia (lasting less than 48 hours), patients might experience symptoms such as lethargy, weakness, irritability, seizures, and coma. On the other hand, chronic hypernatremia (lasting more than 48 hours) can manifest as relatively mild or similar to acute hypernatremia and may progress to obtundation or coma. Additional symptoms may include heightened thirst, increased urinary frequency and volume, as well as nonsecretory diarrhea or vomiting. It is important to note that hypernatremia is linked to higher mortality rates in critically ill and elderly patients [11,12]. In extreme cases of hypernatremia (plasma sodium exceeding 226 mmol/L), fatal ventricular tachycardia may occur [13].

Chronic hypernatremia is managed by administering isotonic saline to hypovolemic patients until their vital signs return to normal. Subsequently, D5W is administered in accordance with the water deficit and losses from stool, sweat, respiration, urinary-free water, and insensible losses. Treatment aims to decrease plasma sodium levels by 8-10 mmol/day. Acute hypernatremia, on the other hand, is addressed by lowering plasma sodium by 1 mmol/L/hour within the initial six to eight hours.

The patient had a documented medical history of major depressive disorder and was admitted to a behavioral health unit due to symptoms of depression and reduced oral intake. Her condition was further complicated by challenges with taking medications, undergoing laboratory tests, and dealing with a urinary tract infection, which ultimately led to tachycardia, dry mucous membranes, and hypernatremic dehydration, with a plasma sodium concentration reaching 191 mmol/L after 12 days. Throughout this period, she remained responsive but lethargic.

Patients with psychiatric conditions may experience hypernatremia as a result of a deficiency in thirst regulation. This can be attributed to three main factors: central nervous system lesions, excessive mineralocorticoid levels, and the side effects of certain medications, such as lithium [14]. Since none of these factors were present in our patient, we can infer that her depression may directly impair her ability to feel thirsty.

The patient was transferred to the intensive care unit to monitor her condition closely. She received a 1-liter bolus of D5W, followed by D5W/1/2 NS at a 150 mL/hour rate. The next day, her lab results revealed a plasma sodium concentration of 178 mmol/L, and the D5W infusion rate was adjusted accordingly. In a case report by Hassan et al., their patient also had a plasma sodium concentration of 191 mmol/L and was treated with 5% dextrose normal saline infusions given at 200 mL/hour for six hours, followed by 150 mL/hour with subsequent adjustments. The sodium level reached 173 mmol/L the following day [15].

While there are no definitive guidelines on the rate of sodium correction for severe hypernatremia, several research studies suggest specific approaches. For chronic hypernatremia, a sodium correction rate between 8 and 10 mmol/day is recommended, whereas for acute hypernatremia, a reduction of 1 mmol/L/hour in the first six to eight hours is advised, with a caution not to exceed a reduction rate of 0.5 mmol/L/hour. Rapid correction of hypernatremia may lead to seizures and cerebral edema, especially in cases of chronic hypernatremia. However, a study by Chauhan et al. did not find evidence linking rapid correction of hypernatremia to increased risk of mortality, seizures, altered consciousness, or cerebral edema in critically ill adult patients with either admission or hospital-acquired hypernatremia [16]. In a case report by Hassan et al., a patient was corrected at a rate of 18 mmol/L in 1 day. In our patient’s case, plasma sodium was corrected at a rate of 13 mmol/L in less than 24 hours without any resulting neurological complications.

## Conclusions

Severe hypernatremia resulting from dehydration in psychiatric patients is an uncommon occurrence, often characterized by an exceptionally high plasma sodium concentration. This can be influenced by factors such as reduced thirst sensation, excessive mineralocorticoid hormones, or side effects of medications. These patients often show withdrawal symptoms and may refuse to take oral medication, eat, or undergo laboratory tests, making it challenging for doctors to diagnose and manage the condition early, so it is crucial for physicians to promptly identify the situation and provide tailored treatment to prevent potential complications. Treatment involves addressing water deficits and losses and administering D5W. Recent research suggests that rapid correction of hypernatremia in these patients may be acceptable.
